# The experience of bearing a child: implications on body boundaries and their link to preterm birth

**DOI:** 10.1186/s12884-023-06203-2

**Published:** 2024-02-05

**Authors:** Nina Spaegele, Julia Ditzer, Mariana Rodrigues, Anat Talmon

**Affiliations:** 1 Department of Psychology, Stanford University, Stanford, CA USA; 2 Faculty of Psychology, Clinical Child and Adolescent Psychology, Technische Universität Dresden, Dresden, Germany; 3 Paul Baerwald School of Social Work and Social Welfare, The Hebrew University of Jerusalem, Mount Scopus, Jerusalem, 91905 Israel

**Keywords:** Body boundaries, Body perception, Pregnancy, Childbirth, Preterm birth

## Abstract

**Background:**

Preterm birth, which occurs when a baby is born before 37 weeks, has enormous implications for public health. It is the leading cause of infant death and mortality in children under the age of five. Unfortunately, the multifaceted causes of preterm birth are not fully understood. One construct that has received increasing attention in women’s transition to motherhood is body boundaries, i.e., the metaphorical barriers that separate the self from the outer, surrounding “not self.” This study aims to examine the role of well-defined and disturbed body boundaries in predicting preterm birth.

**Methods:**

A sample of 655 Israeli pregnant women reported their sense of body boundaries (BBS, as measured by the Sense of Body Boundaries Survey) pre- and postnatally. We performed a General Linear Model (GLM) testing the effect of the BBS total score on the days women delivered before their due date and controlling for whether it was the women’s first child.

**Results:**

Our GLMs controlling for whether it was the women’s first child showed that the BBS total mean exhibited a significant predictive effect on the number of days delivered before the due date (*F*(57,313) = 3.65, *p <* .001).

**Conclusions:**

These results demonstrate heterogeneity in women’s sense of body boundaries during pregnancy and are the first to disentangle a link between disturbed body boundaries and preterm birth. Mediating mechanisms in this relation, e.g., psychosocial stress, as well as clinical implications are discussed in detail.

## Background

Preterm birth, occuring in about 12% of pregnancies worldwide, is the leading cause of neonatal morbidity and mortality. Evidence suggests that the rate of preterm birth may not only be related to physical symptoms, but also to psychological factors [1, 2]. One potential influential factor is a woman’s sense of body boundaries. While body boundaries have been of increasing research interest, it is still unknown whether they are a stable construct over the lifespan or whether they are malleable, and if so what life-events cause them to change.

### The importance of well-defined body boundaries

The boundary of the body defines the self: it separates the self from its surroundings [3, 4] and establishes a clear line between the self and the other [5]. Those boundaries contribute to an individual’s sense of self-sustainability and existence [6], in which their body is considered integral to their self.

Research has, thus far, revealed that the sense of body boundaries differs between individuals [4, 7]. Those who have well-defined boundaries are able to be attentive to their body sensations, to be sensitive to them, and to interpret them in an appropriate way [4]. On the other hand, people experiencing a sense of disrupted body boundaries may find it difficult to identify their body sensations, reflecting alienation from their bodies. This difficulty may manifest, for example, as apathy towards their bodies [8], or as an exaggerated sensitivity to body signals, leading to a sense of threat [9].

The integrity of body boundaries is influenced, for example, by an individual’s self-consciousness [10] and life events [4, 11, 12]. Expanding our knowledge of the role of body boundaries in pregnancy and birth is important as a person’s conceptualization of their physical aspects and their awareness of their body are associated with less engagement in unhealthy prenatal behaviors and decreased postnatal depression [13].

### Body boundaries and pregnancy

As previous studies have indicated the sense of body differentiation to be related to periods of transition [14], the present study explores body boundaries during women’s pregnancy and adjustment to motherhood. In this period of change and adjustment, women may be particularly challenged in terms of their sense of self and sense of their bodies [7, 11]. During pregnancy, the body experience is a reflection not only of the physical changes taking place, but also of the formative process of becoming a mother [15]. These physical and mental changes in pregnancy affect women’s perceptions of their bodies and may impact their sense of body boundaries.

First, women experience drastic physical changes during the course of their pregnancies, both in terms of its appearance and functionality. Facing their transforming body size, shape, and weight, some pregnant women become less aware of their body dimensions [16]. Conversely, pregnancy may increase body awareness through higher connectedness to bodily functions and sensations [13]. In pregnancy, women’s bodies may demand increased attention as physical states, such as hunger and fatigue, are experienced more intensely [13]. Further, pregnant women may be more attuned to their inner physical experiences as a result of knowing that their future child is developing inside them [13, 15]. Pregnancy can thus intensify a woman’s attention to her body and its functions.

In addition, some women experience a new sense of meaning and connection to their bodies due to its ability to create life and their evaluation of its functionality [17]. Others report feeling out of control due to the enormous physical changes taking place in their bodies [18, 19], potentially thus perceiving their bodies as separate from the self.

Further, pregnancy involves the sharing of a woman’s body with another organism. Women’s responses to this condition range from experiencing comfort and pride to feeling invaded [18]. Some women have further reported feelings of confusion regarding their body boundaries and their bodies’ separation from both the fetus and the outside world [20, 21]. These women may be particularly impacted by a sense of disrupted body boundaries. Finally, women’s sense of body boundaries may be impacted by the public nature of pregnancy. While pregnancy is a personal experience, its visibility can make it a very public one, attracting attention [22]. Although some women enjoy this attention, others feel that their bodies have become “public property” [20].

## Body boundaries and preterm births

As is evident, the experience of pregnancy and the transition to motherhood constitute a significant transition in life. Both the physical and mental changes hold the potential to alter women’s perceived body boundaries. Investigating this link further is of importance as psychological factors such as anxiety and depression during pregnancy are associated with slowing growth of the fetus, giving birth to a low birth weight infant, fetal distress, adverse neurodevelopmental outcomes for the child, and preterm birth [1, 2].

Preterm birth, particularly, is of immense clinical relevance. Still, its multifaceted etiology is not fully understood. Physical conditions, such as prior history of pregnancy and abortion, complications of maternal hypertension, fetal growth restriction, premature rupture of the membranes, placenta previa, and abnormal presentation, are associated with preterm birth [23, 24]. However, these risk factors do not adequately predict preterm births. Psychological factors in particular hold potential for further insight, as they have been shown to increase the risk for preterm birth and potentially interfere with women’s ability to maintain a healthy pregnancy [25].

Substantial evidence points to the intricate relationship between psychological factors and preterm birth, yet there remains a substantial gap in our knowledge. An underexplored and compelling factor potentially contributing to preterm birth is the sense of one’s body boundaries as disrupted. This disruption could exacerbate the perceived challenges and stress experienced by expectant mothers. The existing body of literature has already illuminated connections between various body-related experiences and preterm birth. The existing literature has already illuminated connections between various body-related experiences and preterm birth. For example, research has shown that experiences such as body dissatisfaction and shame during pregnancy are linked to heightened risk of preterm birth [18, 24, 25].

Moreover, stress related to body image has been shown to correlate with increased depressive symptoms and anxiety [26], both of which have established links to birth complications [27, 28] and preterm birth [29–32]. Furthermore, during pregnancy, women may experience internally-oriented body experiences, such as a sense of estrangement from their own bodies and a diminished sense of embodiment, making it challenging to adapt to the physical changes that pregnancy entails [11].

These experiences of body estrangement have been positively associated with psychological distress and negatively associated with overall well-being [33]. In a domino effect, cumulative psychosocial stress has been established as a significant risk factor for preterm birth [34, 35]. Therefore, disruptions in the sense of body boundaries may play a critical role in the complex landscape of preterm birth, influencing the mental and physical health of expectant mothers, ultimately impacting the timing of childbirth.

## The current study

In conclusion, the body experience during pregnancy is a specific and particular experience, and yet to date this experience and its implications have hardly been systematically studied. We aim to address this gap in the literature through the present study. It is conceptualized as a two-study design. In study 1, we use a case-control design to investigate whether perceived body boundaries change in pregnant women (T1, T2) as compared to non-pregnant women (T1, T2). In Study 2, we relate antepartum sense of body boundaries to preterm birth.

We hypothesize that perceived body boundaries will undergo significant changes in pregnant women between two time points (T1, T2), with differences observed when compared to non-pregnant women at corresponding time points (T1, T2). Specifically, we anticipate an increase in body awareness and boundary differentiation during pregnancy due to the unique physical and mental changes associated with this life phase. In Study 2, we expect greater disruption or instability in antepartum sense of body boundaries to be associated with an increased risk of preterm birth. This hypothesis is based on the premise that a disrupted sense of body boundaries can exacerbate the challenges and stressors faced by expectant mothers, which, in turn, may adversely affect pregnancy outcomes.

## Materials and methods

### ***Samples and procedure***

**Pregnancy sample.** Participants in Time 1 of the study were 655 pregnant Israeli women. They were recruited via social media (i.e., Facebook, online forums dedicated to the topics of pregnancy and transition to motherhood), and invited to participate in a study being conducted on “The long-term effects of negative childhood experiences on the transition to motherhood.” Participants had to be Hebrew-speaking and at least 18 years old. Of the 655 Time 1 participants, 394 (60%) participated at Time 2 (*M* = 8.54, *SD* = 3.33, range 3–21 weeks postpartum).

We conducted a series of analyses to examine whether there was a pattern of selective attrition. The two groups did not differ in number of children *t*(653) = 0.35, *p* = .732 or family status, *t*(3) = 2.47, *p* = .484. However, the groups did differ in age *t*(651) = 3.01, *p* = .031, Cohen’s d effect size = 0.24; level of education *t*(626) = 4.16, *p* < .001, Cohen’s d effect size = 0.33; and socio-economic status, *t*(2) = 11.03, *p* = .004. Time 2 respondents were slightly older (*M* = 30.96, *SD* = 4.68) than dropouts (*M* = 29.83, *SD* = 4.70). They also reported being slightly more educated than dropouts (*M* = 16.19 years, *SD* = 2.27 for the respondents; *M* = 15.37 years, *SD* = 2.64 for dropouts). Further, a higher number of Time 2 respondents than dropouts reported an income higher than the average income in Israel (*n* = 126, 32.1% for respondents, *n* = 58, 22.5% for dropouts). The following analyses in this study included the 394 women who participated in both the Time 1 and Time 2 assessments, with an average time between T1 and T2 responses was 19.14 weeks.

Demographics are illustrated in Table 1. At Time 1, 7.4% of the participants (*n* = 29) were currently in the first trimester of their pregnancies ( < = 13 weeks); 34.5% (*n* = 136) were in the second trimester (14–26 weeks); and 58.1% (*n* = 229) were in the third trimester ( > = 27 weeks). Fifty women (12.7%) reported having undergone fertility treatment, and 17% (*n* = 67) reported high risk pregnancies. Approximately half the women (*n* = 202, 51.3%) were pregnant with their first child.

**Table 1:** Demographics for pregnancy sample and control group

Sample	Age	SES					Years of Education
		Far Below Average	Below Average	Average	Above Average	Far Above Average	
Pregnancy Sample	30.96 (4.68)	-	41.22%	26.72%	32.06%	-	16.19 (2.27)
Control Group	31.51 (6.24)	22.03%	31.06%	18.51%	25.69%	3.74%	16.04 (2.93)

**Control group.** For comparison, a control group of *N* = 337 Hebrew-speaking Israeli women at least 18 years of age were recruited. Fifteen of these participants had to be excluded as they completed questionnaires in under seven minutes, indicating that these participants may not have given sufficient attention to the questionnaire items. As a result of the little time spent on the questionnaires, the responses likely do not accurately reflect the participants’ true perceptions and experiences, which could compromise the reliability of our results. This resulted in a final sample size of *n* = 322. Participants were recruited through social media platforms. For their participation, participants were given the opportunity to enter a gift card lottery. They completed questionnaires in randomized order through Qualtrics Research Software.

Demographics of the control group are illustrated in Table 1. 279 women participated in the study at T1 but dropped out before the T2 assessments.

## Measures

*Sense of disrupted body boundaries.* Women’s sense of body boundaries was assessed at Time 1 and Time 2 by the Sense of Body Boundaries Survey (BBS) [26]. The BBS is a 17-item scale, consisting of two subscales: the Barrier subscale, measuring the individual’s sense of physical separateness from their surroundings (e.g., “My feeling of physical separation from the environment is rather vague,” “I don’t feel strictly separated from the surrounding reality”) and the Permeability subscale, measuring the individual’s sense of body vulnerability (e.g., “I feel that my body is susceptible to outer influences,” “Sometimes I imagine my body as a wide-open window”). Participants indicated the extent to which the statement described their body experience on a five-point Likert-type scale, with scores ranging from 1 (*definitely don’t agree*) to 5 (*strongly agree*). Mean scores were used, with higher scores in the Barrier and Permeability subscales representing higher levels of a sense of disrupted body boundaries. Validity of scores on the BBS was supported by its positive correlation with the Body Self Questionnaire [4]. Reported internal consistency and test–retest reliability for scores on the BBS were 0.87 and 0.68–0.83 respectively [26]. The scale was originally written in Polish. It was translated into Hebrew using a synthesis of multiple independent translations.

### Statistical analyses

#### Study 1

First, we examined the descriptives for the BBS average score as well as its two subscales in the pregnancy sample and control group separately at T1 and T2. We then used paired samples *t*-tests to test whether the BBS average or its subscales’ scores changed significantly between T1 and T2 in the group of pregnant women, and then in the control group.

#### Study 2

Using the sample of pregnant women, we first calculated bivariate Pearson correlations between the BBS total average, its two subscales, and the number of days women gave birth before their due date. Next, we performed a General Linear Model (GLM) testing the effect of the BBS total score on the days women gave birth before their due date and controlling for whether it was the women’s first child. We repeated these analyses with the BBS subscales.

## Results

### Study 1

In pregnant women, the average score of the BBS was 1.82 (*SD =* 0.56) at T1 and decreased to 1.62 (*SD* = 0.47) at T2. The difference between T1 and T2 was significant (*t*(321) = 6.93, *p* < .001). Looking at the BBS subscales, we found that the average scores in both subscales significantly decreased between T1 (BBS subscale “Barrier” *M =* 1.59, *SD* = 0.60; BBS subscale “Permeability” *M =* 1.94, *SD =* 0.55) and T2 (BBS subscale “Barrier” *M =* 1.39, *SD* = 0.51; BBS subscale “Permeability” *M =* 1.75, *SD* = 0.50) in pregnant women (“Barrier”: *t*(321) = 6.15, *p* < .001 two-sided; “Permeability”: *t*(321) = 6.43, *p* < .001 two-sided).

**Fig. 1:**
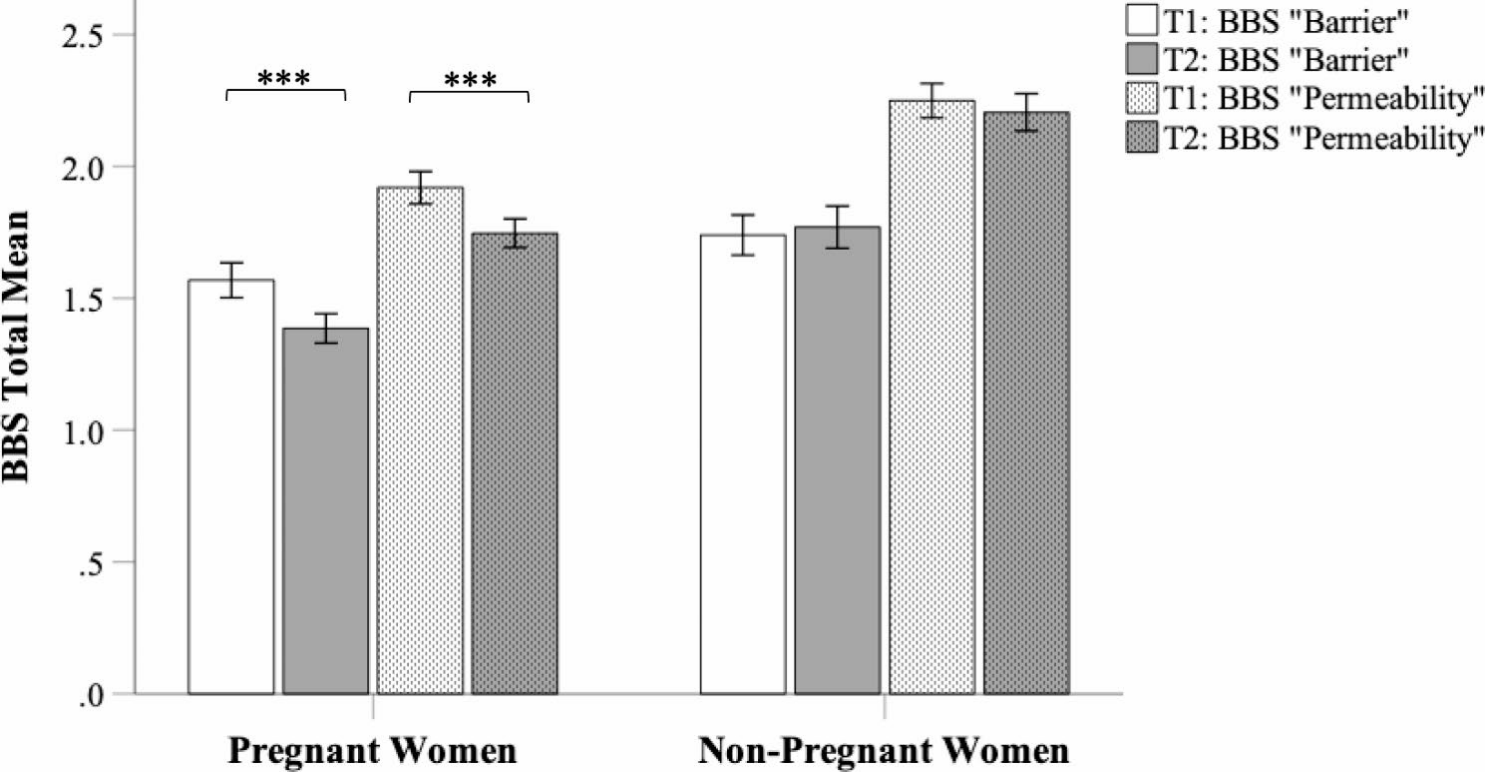
T1 and T2 mean score of sense of body boundaries survey subscales in pregnant vs. non-pregnant women. *Note: BBS* Body boundaries survey. ***p < .001

In the control group, the average score of the BBS was 1.87 (*SD* = 0.70) at T1 and increased to 2.01 (*SD* = 0.68) at T2. For the overall BBS score, the difference between T1 and T2 was significant (*t*(304) = -22.27, *p* < .001). However, looking at the BBS subscales, we found that the average scores in both subscales did not significantly change between T1 (BBS subscale “Barrier” *M =* 1.77, *SD =* 0.70; BBS subscale “Permeability” *M* = 2.26, *SD* = 0.60) and T2 (BBS subscale “Barrier” *M =* 1.77, *SD* = 0.71; BBS subscale “Permeability” *M =* 2.2, *SD* = 0.63) in non-pregnant women (“Barrier”: *t*(304) = -0.90, *p =* .369 two-sided; “Permeability”: *t*(304) = 1.44, *p* = .152 two-sided). These results are depicted in Fig. 1.

### Study 2

The bivariate Pearson correlation between the BBS total mean and the number of days women gave birth before due date was non-significant (*r =* .05, *p =* .312) and so were both the correlations with the BBS subscale “Barrier” (*r =* .07, *p =* .149) and “Permeability” (*r =* .03, *p =* .509).

**Fig. 2:**
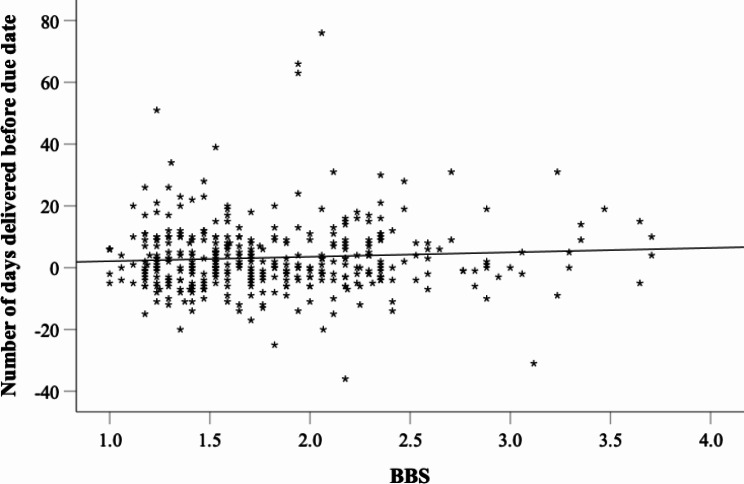
Scatter plot depicting the number of days women gave birth before due date depending on sense of body boundaries survey. *Note: BBS* Sense of body boundaries survey total mean

However, as our GLMs controlling for whether it was the women’s first child have shown, the BBS total mean exhibited a significant predictive effect on the number of days women gave birth before the due date (*F*(57,313) = 3.65, *p <* .001, *partial η*^*2*^ = 0.4, *R*^*2*^_*whole model*_ = 0.4 (Adjusted *R*^*2*^ = 0.29)). Figure 2 depicts the number of days women gave birth before their due date as a function of the BBS total mean.

Moreover, both BBS subscales as well as their interaction significantly predicted the number of days women gave birth before the due date (Barrier: *F*(17,221) = 2.17, *p <* .001, *partial η*^*2*^ = 0.14, Permeability: *F*(37,221) = 2.84, *p <* .001, *partial η*^*2*^ = 0.32, Barrier x Permeability: *F*(93,221) = 2.15, *p <* .001, *partial η*^*2*^ = 0.48, *R*^*2*^_*whole model*_ = 0.6 (Adjusted *R*^*2*^ = 0.33)), despite controlling for whether it was the women’s first child.

## Discussion

As we hypothesized and demonstrated in our study, women perceive body boundaries diversely as they experience pregnancy. Even though prior research has aimed to elucidate preterm birth predictors and establish a connection between psychological factors, much remains unclear on the role of the sense of body boundaries. To our knowledge, this study is the first to explore a possible link between women’s sense of disrupted body boundaries and preterm birth. While no significant connection was found, our results suggest that the number of pregnancies might alter women’s sense of body boundaries and its relation to preterm birth. These results underscore the importance of further investigation into the complex relationship between body boundaries and pregnancy outcomes, particularly preterm birth. The understanding that the number of pregnancies can impact a woman’s perception of her body boundaries represents a novel insight and suggests that there may be underlying factors contributing to the intricate experiences of expectant mothers.

As mentioned above, previous studies have associated preterm birth with psychological factors and adverse life experiences. Behrman & Butler [27], for instance, have linked preterm birth with one’s experiences of severe life events, stress exposure, perceived social support and control, maternal anxiety, and racism. However, the role of the sense of body boundaries has not yet been proven to increase the risk of preterm birth, though evidence suggests there to be an indirect link between them. As such, prior research implies that DDB likely increases stress levels and challenges during the transition to motherhood, thus possibly increasing the risk for stress-related pregnancy complications such as preterm birth.

Brubaker & Wright [28] stated that women’s sense of autonomy is often influenced by body experiences during pregnancy. As such, Johnson et al. [20] and Schmied & Lupton [21] have argued that a sense of disrupted body boundaries may impact some women who, through interviews, reported confusion regarding their body boundaries and body-baby-environment separation. Further, Talmon et al. [7] examined body-differentiation and its effect on adjustment to motherhood and concluded that the transition to motherhood presents challenges that cannot possibly be addressed when both the body and the self are perceived as vulnerable and disrupted. Moreover, women with “undifferentiated” patterns between the body and self-reported low levels of body agency, increased mother-infant bonding difficulties, high levels of body estrangement, and a more challenging adjustment to motherhood.

In the present study, we explored the role of women’s sense of body boundaries during women’s pregnancy and adjustment to motherhood. In particular, we investigated disrupted body boundaries as a potential risk factor for predicting preterm birth. Using a two-study design, our results show that in pregnant women, the average of BBS decreased significantly from T1 to T2 (general subscales) and increased in the control group. Further, our GLMs controlling for whether it was the woman’s first child showed that the BBS total means exhibited a significant predictive effect on the number of days women gave birth before the due date. These results demonstrate heterogeneity in women’s sense of body boundaries in the transition to motherhood. Thus, our study paves the way for future research while bringing light to a link between a sense of disrupted body boundaries and preterm birth.

### Body boundaries and pregnancy

Prior research has examined women’s experiences with pregnancy and their bodies. The body experience during pregnancy is a particular experience [11] that has yet to be thoroughly studied. Our study’s results expand on current literature about the diverse experiences one may undergo during pregnancy.

Moreover, as diverse as women’s experiences of their bodies during pregnancy are, they may reflect their reactions to concrete physical changes as well as self-representations during the transition to motherhood [11]. Prior research has explored women’s sense of embodiment during pregnancy regarding physical and psychological changes [29], appearance, and functionality [15]. For instance, during pregnancy, the boundaries between the self and the other undergo significant changes [30], and the dichotomy between self and the outside world is broken down [31]. As such, Hodgkinson et al. [18] have argued that women might react to the sharing of their bodies with diverse feelings, both positive and negative. Such feelings might range from a feeling of comfort to one of being invaded, penetrated, and alienated from their bodies [18]. Therefore, the variety in feelings and the unique feature of pregnancy may cause women to experience a sense of disrupted body boundaries. Evidence has shown that women’s reported experiences include a feeling of confusion about their body boundaries and their bodies’ separation from both the fetus and the outside world [20, 21].

Our results expand the literature on the aforementioned connection between the diversity of women’s sense of body boundaries, the uniqueness of pregnancy, and the understanding of the wide range of implications related to the body experience during this period by showing a significant decrease in the average score of the BBS among participants before and after birth. This connection reaffirms the findings that pregnancy is a complex period and is often associated with changes in women’s perceptions of body boundaries. Moreover, other factors such as poor socioeconomic conditions and child maltreatment have also been associated with the development of a sense of disrupted body boundaries and altered body image as well as an increase in the risk of improper health behaviors, inadequate access to prenatal care, promoting the risk of preterm birth (Dolatian et al., 2018).

### Body boundaries and preterm birth

To our knowledge, no previous studies have investigated women’s sense of body boundaries as predictors of preterm birth. Our aim to explore this possible connection was constructed through previous findings showing that (a) physical and mental changes hold the potential to alter women’s perceived body boundaries, and (b) psychological factors are associated with pregnancy outcomes, such as heart rate of the fetus, birth weight, fetal distress, congenital malformations, and preterm labor [1, 2]. Further, a sense of disrupted body boundaries has been found to possibly add to a mother’s perceived challenges and stress. As such, research has shown that women have reported a sense of loss of control as a result of the enormous changes taking place in their bodies [18, 19] as well as the feelings of fear, violent penetration and alienation within their pregnancy [30]. Concurrently, psychological stressors are associated with an increased risk for preterm birth [25], thus leading to the hypothesis that a sense of disrupted body boundaries may predict preterm birth.

Unexpectedly, our study’s findings did not demonstrate a significant connection between women’s reporting on their sense of body boundaries and their due dates. This might be attributed to the fact that a sense of body boundaries may vary from person to person, as it is a subjective, cognitively-emotional experience [32–34], that is constantly being remodeled and reworked through our relations with the world around us [35]. Thus, one’s sense of body boundaries may change throughout life and has many determinants that might affect how one might perceive their body in connectedness to the self and the environment. This subjective and malleable perception of body boundaries might be the reason why our findings did not demonstrate a significant connection, as pregnancy could be hypothesized to have contributed to an improvement in some women’s sense of body boundaries.

However, the results did exhibit a significant predictive effect on the number of days women gave birth before the due date when controlling for whether it was the woman’s first child, looking at both BBS subscales as well as their interaction. These findings are consistent with prior research examining the role of pregnancies and body perception. Talmon & Gizburg [11], for instance, assessed the psychological representations of the body experience during pregnancy. The study states that pregnant women may experience body dissatisfaction [18]; feel ashamed of their pregnant bodies [36]; as well as suggests changes in one’s sense of “feeling at home” in their own body, due to the perpetually changing pregnant body [37].

Moreover, with the presence of the developing fetus during pregnancy, a woman may feel as if her sense of personal space is violated and experience alienation from her body [11]. Upton & Han [38] have also suggested that women might feel as if they are losing themselves in their bodies during pregnancy. In addition, evidence shows that the disruption of women’s sense of body boundaries during pregnancy may reach its climax during the birth [31, 39], thus providing a link between pregnancy and possible changes in women’s sense of body boundaries.

As such, our findings positively contribute to the current research by expanding on the relationship between a sense of disrupted body boundaries and pregnancy as well as providing insight into the possible high-risk factors of preterm birth. Moreover, our study establishes a connection between previously studied factors for premature birth as well as shows a significant relationship linking DDB and the number of days women gave birth before their due date. Finally, our research demonstrates diversity in women’s sense of body boundaries in the transition to motherhood and is a pioneer in examining a link between disrupted body boundaries and preterm birth.

### Clinical implications

Previous studies have found that pregnancy is a unique experience and may affect women’s sense of body boundaries [40, 41]. Concurrently, evidence has also associated altered body image and prematurity [42]. In our study, we set out to explore the diverse ways in which women perceive their body boundaries during pregnancy and its potential impact on preterm birth. While our findings did not reveal a statistically significant connection, they shed light on a possible link between the number of pregnancies and the alterations in women’s sense of body boundaries, along with its influence on preterm birth. In combination, these findings indicate that expanding on the literature regarding body boundaries and preterm birth is needed as it provides the knowledge and tools to address and assess perceptions of disrupted body boundaries within clinical practice.

In fact, the assessment of a sense of disrupted body boundaries during prenatal visits could be considered as clinicians care for women in the pregnancy period. This evaluation could help healthcare professionals better understand and address the unique challenges that pregnant individuals may face. Addressing women’s sense of body boundaries also allows professionals to provide women with psychoeducation and treatment that may reduce the risk of pregnancy complications such as preterm birth.

Moreover, treatment within clinical care can consist of integrative interventions focused on the body-mind-environment connection (i.e. Mindfulness-Based Stress Reduction techniques), which aim to address possible disruption in the balance of how people perceive the self in relationship with their bodies and the environment [43]. Talmon & Ginzburg [11] highlight the importance of verbalization and processing of one’s feelings about their body. Thus, addressing one’s perceptions of their body is essential when aiming to defragment the broken “self” and establish a well-defined “body self” within the therapeutic setting.

In conclusion, to further inform clinical practice, future studies should explore risk and resilience factors in this relation to better understand and modify critical factors in high-risk pregnancies and preterm birth.

### Strengths and limitations

Our study has several strengths. First, the large sample size allowed us to collect valuable and diverse data. Further, having a control group was essential as we had greater comparability as well as more information regarding the potential differences in response between T1 and T2 participants and the control group. Our study is also the first to examine the link between women’s sense of body boundaries and preterm birth as well as pave the way for future research.

However, some limitations of our study include the fact that the utilized measure was not originally written in Hebrew. The scale was originally written in Polish and translated into Hebrew using a synthesis of multiple independent translations. The potential influence of culture and language must be taken into account by future studies in this field, even though the BBS is a valid and reliable measure [26].

Moreover, although our results show heterogeneity within women’s sense of body boundaries, there is a need to consider factors such as race, ethnicity, income and education level, sexual identity, and/or other psychosocial factors as they may play a role in women’s perceptions and reporting on body boundaries. Moreover, our sample consists of only cisgender women. Further research is needed regarding sexual minorities’ experiences.

In addition, it is important to note that the sample sizes in the current study were constrained by the data’s origin within the scope of another project, which could have limited our ability to detect some of the hypothesized effects. We recognize the potential limitations in statistical power, and we emphasize the importance of future replication studies to bolster and validate the current findings.

Finally, even though our results do show variability between pre and post-birth data, there is still a need to examine changes in their sense of body boundaries women might experience within a longitudinal approach. Women’s perceptions of their bodies must be analyzed by considering levels of changes or stability within periconceptional, pregnancy, and postpartum periods. Further, it is important to investigate whether pregnancy can be known to improve women’s sense of body boundaries. For instance, recruiting participants in clinical settings, which can facilitate the conduction of follow-ups during pregnancy and postpartum would be convenient. Thus, other potential mediators that may affect women’s sense of body boundaries could be addressed and examined multiple times within the pregnancy period.

As such, future research on body boundaries and pregnancy should explore the unique nature of women’s experiences and environment, controlling for whether or not age, educational level, family status, life events, and average income have a higher predictive value of a sense of disrupted body boundaries during pregnancy. Further, studies should further examine a possible connection on whether a sense of disrupted body boundaries might add to a mother’s perceived stress and challenges. Lastly, an additional topic worthy of further research relates to body boundaries and people who give birth, to address a current literature gap regarding transgender men, non-binary, and queer pregnant people who experience uniqueness within their pregnancies themselves.

## Conclusion

Women’s sense of body boundaries has been understudied as a possible predictor for preterm birth. Although prior research suggests a connection between the effect of psychosocial factors, how women perceive the challenges of motherhood, and preterm birth, little has been studied about the connection between women’s sense of body boundaries and preterm birth. Our study’s findings show (a) heterogeneity in women’s reportings of their body boundaries and (b) suggest a connection between women’s sense of body boundaries and preterm birth. This connection is demonstrated by the fact that a disrupted sense of body boundaries predicted a higher number of days a woman’s child was born before the due date. Moreover, future research may use these findings to further examine the role of women’s sense of body boundaries in predicting preterm birth when incorporating other potential mediators and looking at a wider scope of populations. Finally, our results also enable clinical settings to consider the importance of care for pregnant populations in understanding and modifying critical factors in high-risk pregnancies and preterm birth.

## Data Availability

The datasets generated and/or analyzed during the current study are not publicly available due to the sensitive nature of the questions asked but are available from the corresponding author on reasonable request.
